# 17α-Hydroxylase/17,20-lyase Deficiency (17-OHD): A Meta-analysis of Reported Cases

**DOI:** 10.1210/clinem/dgae773

**Published:** 2024-11-06

**Authors:** Annabelle L Willemsen, David J Torpy, Sunita M C De Sousa, Henrik Falhammar, R Louise Rushworth

**Affiliations:** School of Medicine, University of Notre Dame Australia, Sydney 2010, Australia; Endocrine and Metabolic Unit, Royal Adelaide Hospital, Adelaide 5000, Australia; Endocrine and Metabolic Unit, Royal Adelaide Hospital, Adelaide 5000, Australia; South Australian Adult Genetics Unit, Royal Adelaide Hospital, Adelaide 5000, Australia; Adelaide Medical School, University of Adelaide, Adelaide 5005, Australia; Department of Molecular Medicine and Surgery, Karolinska Intitutet, Stockholm 171 77, Sweden; Department of Endocrinology, Karolinska University Hospital, Stockholm 171 77, Sweden; School of Medicine, University of Notre Dame Australia, Sydney 2010, Australia

**Keywords:** congenital adrenal hyperplasia, CYP17A1 deficiency, disorder of sexual development

## Abstract

**Purpose:**

Homozygous pathogenic variants in the *CYP17A1* gene result in defective activity of the steroidogenic enzymes 17α-hydroxylase/17,20-lyase resulting in the clinical syndrome 17-OHD characterized by hypertension, hypokalemia, and disorders of sexual development. Pathogenic variants of *CYP17A1* lead to complete or partial loss of enzymatic activity and clinical presentations of varying severity. This study aimed to examine relationships between *CYP17A1* genotype and clinical presentation in a global cohort.

**Methods:**

We searched PubMed and Scopus for case reports and cohort studies reporting clinical data on patients with 17-OHD published between 1988 and 2022. Of 451 studies, 178 met inclusion criteria comprising a total of 465 patients. We pooled patient data and examined associations between causative variants and their clinical presentations.

**Results:**

There were 465 unique patients with a mean age of 18.9 (9.0) years, 52.5% (n = 244) were XY and 6.4% (n = 29) were phenotypically male. Homozygous variants were seen in 48.0% (n = 223) of patients. Common clinical presentations were hypertension (57.0%, n = 256), hypokalemia (45.4% n = 211), primary amenorrhea (38.3%, n = 178), cryptorchidism (15.3%, n = 71), and atypical genitalia (14.2%, n = 66). Frequently occurring variants included p.Y329Kfs (n = 86), p.D487_F489del (n = 44), and p.W406R (n = 39). More severe variants, such as p.Y329Kfs, were associated with hypocortisolism (*P* < .05), combined hypokalemia and hypertension (*P* < .01), and disordered sexual development (*P* < .01).

**Main conclusion:**

17-OHD is a rare, frequently misdiagnosed disease. Male patients are typically diagnosed earlier because of genital dysplasia associated with less severe variants, whereas female patients are typically diagnosed later from primary amenorrhea and hypertension. Patients presenting with disordered sexual development and hypertension should be investigated for 17-OHD.

Congenital adrenal hyperplasia refers to a group of autosomal recessive conditions affecting cortisol synthesis, the most common of which is 21-hydroxylase deficiency (1). By comparison, only approximately 1% of cases of congenital adrenal hyperplasia have 17α-hydroxylase/17,20-lyase deficiency (17-OHD) from defects in the *CYP17A1* gene (2). Located on chromosome 10q24.3, the *CYP17A1* gene encodes for the steroidogenic enzyme 17α-hydroxylase/17,20-lyase (17-OH) (3). 17-OH is expressed in the adrenal glands and gonads and is a branch point in cortisol and sex hormone synthesis (4). This enzyme catalyses 2 distinct steps in steroidogenesis: 17α-hydroxylation of pregnenolone and progesterone to 17α-hydroxylated products and 17,20-bond cleavage of these products to the C19 androgen precursors, dehydroepiandrosterone and androstenedione (Fig. 1) (5).

**Figure 1.**
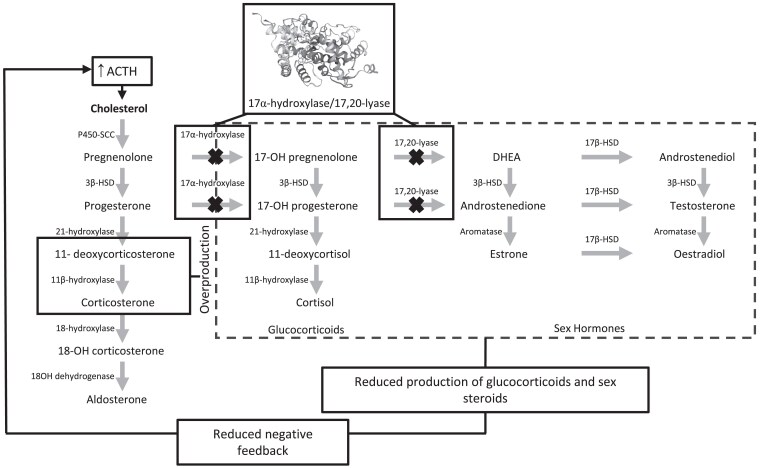
Adrenal steroidogenesis. Enzymes are shown next to arrows indicating each enzymatic conversion. Crosses indicate points of adrenal steroidogenesis pathway impacted by 17α-hydroxylase/17,20-lyase deficiency. Protein structure of 17α-hydroxylase/17,20-lyase is illustrated with boxes connecting it to its points of enzymatic conversion. The dashed box indicates the implicated hormones with reduced production in 17-OHD. Reduced synthesis of both glucocorticoids and sex steroids reduces negative feedback to the pituitary, in turn increasing ACTH release and stimulating the production of mineralocorticoids. Key hormones of interest due to their overproduction in 17-OHD are 11-deoxycorticosterone and corticosterone (indicated by a box). With deficiency of CYP17A1 enzymes 17α-hydroxylase and 17,20-lyase, accumulation of the mineralocorticoid aldosterone contributes to hypertension and hypokalemia. Despite loss of 17α-hydroxylase function and resultant hypocortisolemia, accumulation of deoxycorticosterone and corticosterone prevents glucocorticoid deficiency; thus, few patients experience adrenal crises. Loss of 17,20-lyase function results in impaired sex steroid synthesis with resultant undervirilization and disordered sexual development. Abbreviations: DHEA, dehydroepiandrosterone; HSD, hydroxysteroid dehydrogenase; SCC, side chain cleavage.

More than 100 variants of the *CYP17A1* gene have been discovered including missense variants, single-base deletions, larger deletions, and insertions (5). Variants often cluster within different ethnic groups with the p.Y329k*fs variant most common in Chinese patients and p.W406R and p.R362C in Brazilian patients (6). Pathogenic variants either result in combined or isolated deficiency of the 17α-hydroxylase or 17/20-lyase portions of the protein (3). Most variants cause complete combined 17α-hydroxylase/17,20-lyase deficiency, whereas some result in partial enzyme deficiency (7).

Loss of the encoded 17-OH protein impairs steroid hormone biosynthesis by blocking production of cortisol and sex steroids and causing accumulation of mineralocorticoids and progesterone (Fig. 1). Excessive production of 11-deoxycorticosterone leads to the development of hypertension and hypokalemia, whereas decreased production of sex steroids results in undervirilization of external genitalia in 46,XY patients and primary amenorrhoea and an absence of breast development in 46,XX patients, as well as reduced or absent androgen stimulated hair growth in all individuals (8, 9).

There is limited information on the phenotype of individuals with 17-OHD in relation to genotype, clinical findings, and steroid levels (10, 11). Pooled data from a number of small studies have shown that a combination of serum progesterone, 11-deoxycorticosterone, cortisol, and genital phenotype is predictive of the severity of enzyme impairment (10). This is supported by results from an analysis of 150 46,XY patients that concluded that a combination of serum cortisol and genital phenotype could distinguish between partial and severe 17-OHD (10).

The aim of this study was to enhance the understanding of 17-OHD by analyzing a large sample of pooled data from reported cases and investigating genotype-phenotype relationships in affected individuals.

## Methods

### Case Identification

**Figure 2.**
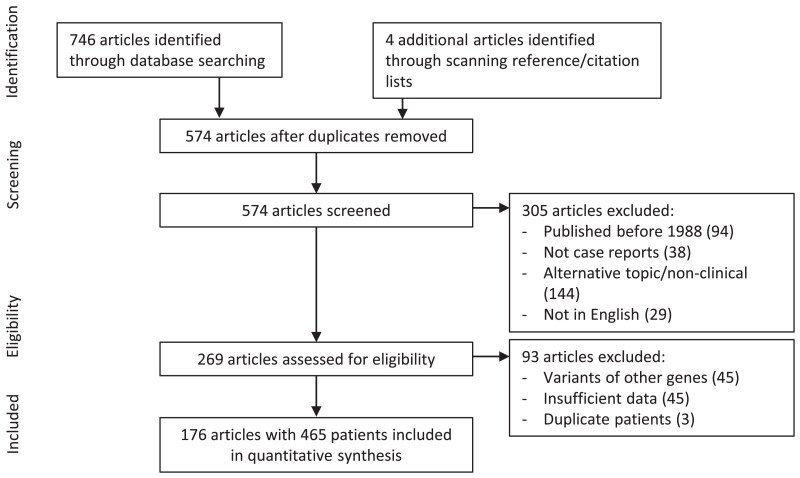
PRISMA flowchart outlining the process of study identification and selection.

Reports on patients with 17-OHD were identified by systematic searches of PubMed and Scopus databases conducted up to August 6, 2022. The broad search term, “CYP17A1 deficiency” was used. These identified 716 case reports with a further 4 articles identified by scanning reference and citation lists (Fig. 2) (12). The Preferred Reporting Items for Systematic Reviews and Meta-Analyses guidelines were followed (13). Quality assessment tools such as the Newcastle-Ottawa Scale or CONSORT checklist were not used because they were not developed for case reports or series (14, 15).

After removal of duplicates, 556 articles were assessed for eligibility. Only articles published after 1988, corresponding to the first report of a 17-OHD variant, were considered eligible for inclusion (16). Articles not in English or without clinical data were excluded. After exclusions, there were 259 articles available for further assessment. A further 88 articles were excluded because of variants involving genes other than *CYP17A1*, insufficient data, or patient duplications. At the end of this process, 467 unique patients from 176 articles remained. Two patients were excluded because of concerns regarding the accuracy of the diagnosis, leaving 465 individual cases for analysis.

### Data Collection

Each patient report was examined for relevant data on genetic variant, nationality, age, karyotype, phenotypic sex, body mass index, blood pressure, serum potassium, pubertal development, and blood hormone levels. Where the same patients were presented in more than 1 publication, data were collected from both articles, if applicable. All data were entered according to a unique patient identifier into an Excel spreadsheet (Excel 2019).

### Data Management

For the purposes of analysis, age was classified into the following groups: <13, 13-18, and >18 years. Hypertension was classified as present if this was reported as such or if a systolic blood pressure level was above 140 mm Hg for patients aged more than 12 years, or according to age related normal levels in younger children (17). Where case profiles did not mention blood pressure, normotension was assumed. In situations where more than 1 blood pressure level or a range was reported, the mean was taken as the value for that patient.

Hypokalemia was classified as present if either hypokalemia was reported or if the potassium level was below 3.5 mmol/L. Tanner staging was collected for pubic hair in 46,XY individuals aged ≥16 years and 46,XX individuals aged ≥14 years. Breast development was classified into 3 groups (none, incomplete, complete) among 46,XX individuals aged 14 years or more at the time of reporting. A combined measure of incomplete/complete or no pubertal development was produced by incorporating breast and pubic hair scores in XX patients and using pubic hair scores in 46,XY patients. Reported “sexual infantilism” was not used due to noncorrespondence of this term with Tanner scores in some patient reports.

Ethnicity was based on the country of origin for each reported case and was considered by country and grouped according to regions.

Genetic variants were labelled according to protein levels, reflecting the historical literature (18). If a published case reported that genetic testing was “positive” but only 1 variant was reported without mention of zygosity; this was taken to mean homozygosity.

### Hormone Concentrations

Serum hormone concentrations differed according to reported units of measurement across case reports but were converted to SI units for analysis. Hormone levels were considered both as continuous variables (reported as mean [SD]) and as categorical variables using reference ranges from the Royal Australasian College of Pathologists and Mayo Clinic, where appropriate (19, 20). Missing values were common and patients with missing information were excluded case-wise from specific analyses. Where available, the following hormones were collected for each patient: aldosterone (pmol/L), ACTH (pmol/L), cortisol (nmol/L), FSH (mIU/mL), LH (mIU/mL), estradiol (pmol/L), progesterone (nmol/L), testosterone (nmol/L), 17-hydroxyprogesterone (nmol/L), dehydroepiandrosterone-sulphate (nmol/L), androstenedione (nmol/L), plasma renin activity (ng/mL/h), corticosterone (nmol/L), 11-deoxycorticosterone (nmol/L), and 11-deoxycortisol (nmol/L). Where multiple values were recorded for cortisol and ACTH, a morning or 8 Am value was taken as the most appropriate measurement for that patient. Categories for each relevant hormone were undetectable, low, normal, or high based on reference ranges. Hypocortisolaemia was classified as a serum cortisol <200 nmol/L. Where hormone levels are virtually undetectable until after puberty (FSH, LH, estradiol, progesterone, testosterone, dehydroepiandrosterone-sulphate, and androstenedione), the same criteria were used as those for pubertal assessment (≥14 46,XX and ≥16 46,XY).

### Variant Classification

Genetic variants were classified as either null or non-null depending on the expected change to the protein product (complete abolishment vs reduced enzymatic function). Noting the limited availability of enzymatic assays, we referred to the American College of Medical Genetics and Genomics guidelines to define null variants as nonsense, frameshift, and initiation codon variants, single, or multiexon deletion, and variants involving the canonical ±1 or 2 splice sites (21). Non-null variants encompassed missense variants and in-frame deletions/insertions.

### Genotype-phenotype Analysis

Relationships between variant severity, hormone levels, and physical findings on presentation were investigated using SPSS and GraphPad Prism (22, 23). *T*-tests and ANOVA were used to assess the differences in distribution of continuous variables and Chi-square tests were used to determine the significance of differences in the distribution of categorical variables. Heat maps were constructed to represent the associations between the severity of genetic variants and the main phenotypic features (hypertension, hypokalemia, and disordered sexual development [DSD]) according to variant severity and the most common genotypes. Where appropriate log transformation was used for continuous variables. A *P*-value of <.05 was considered significant.

### Role of the Funding Source

This study received no direct funding. Salaries of some investigators were supported by unrelated grants as declared.

## Results

This systematic review identified 465 unique patients with 17-OHD of whom 203 (43.7%) were 46,XX, 244 (52.5%) were 46,XY, and the remaining 18 (3.9%) were XXY, XO, X del (X)q12 SRY positive, XO mosaic, or reported as “unknown sex.” The majority (90.8%, n = 422) identified as female, whereas 6.2% (n = 29) were male. Consanguinity was reported in 24.9% (n = 116) and same variant homozygosity in 48.0% (n = 223).

**Figure 3.**
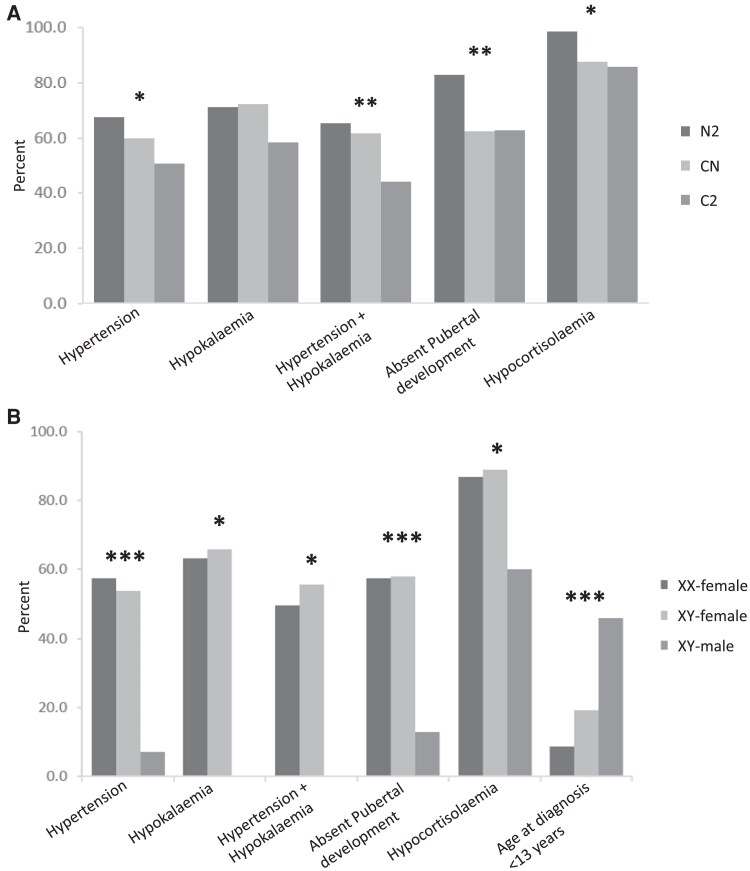
Prevalence of key clinical features in 465 patients with 17α-hydroxylase/17,20-lyase deficiency. (A) Prevalence of key clinical features within variant classifications (N2, NC, and C2). (B) Prevalence of key clinical features and diagnosis before age 13 years within combined gender-karyotype classifications (XX female, XY female, XY male). Significance: *P < .05, **P < .01, ***P < .001. Abbreviations: C, change in sequence; N, null.

The mean age at diagnosis was 19.0 (9.0) years (range, 0-67 years). Sixty-nine (14.8%) patients were <13 years, 143 (30.8%) were aged 13 to 18 years, and 233 (50.1%) were aged >18 years. Males were younger than females at diagnosis (13.0 [10.4] and 19.4 [8.8] years, respectively [*P* < .01]) and patients with a Y chromosome were younger than non-Y patients (17.3 [8.6] and 20.7 [8.7] years, respectively [*P* < .001]). More XY males were diagnosed before age 13 years (45.8%, n = 11) than XY females (19.1%, n = 41) and XX females (8.7%, n = 17) (*P* < .001) (Fig. 3B).

Of the 358 (77.0%) patients with a reported ethnicity, 38.9% (n = 181) were from Asia, 14.6% (n = 68) were from Central or South America, and 12.5% (n = 58) were from the Middle East. Countries with the largest representations were China (29.9%, n = 139), Brazil (12.5%, n = 58), and Turkey (6.5%, n = 30).

### Clinical Presentations

Primary amenorrhea was the most common presenting problem (38.3%, n = 178), followed by cryptorchidism (15.3%, n = 71) and atypical genitalia (14.2%, n = 66) (Table 1). Two patients (0.4%) presented with an adrenal crisis.

**Table 1.** Demographic profile of 465 patients diagnosed with 17α-hydroxylase/17,20-lyase deficiency

Demographics	%	n	Mean	SD	Range
Age at diagnosis (y)		445	19.0	9.0	0-67
Height (cm)		209	161.2	19.3	53-191
Weight (kg)		183	53.3	18.7	2.6-141
BMI		183	20.3	4.8	12.0-46.0
XY status					
XX	43.7%	203			
XY	52.5%	244			
Other	1.3%	6			
Unknown	2.6%	12			
Gender					
Female	90.8%	422			
Male	6.2%	29			
Unknown	3.0%	14			
Consanguinity	24.9%	116			
Blood pressure					
Blood pressure (mm Hg)					
Systolic		354	149.7	25.1	90-240
Diastolic		354	98.6	18.4	50-160
Hypertension*^a^*	57.0%	265			
Hypertensive complications*^b^*	6.7%	31			
Potassium					
Serum potassium (3.5-5.2 mmol/L)		284	3.2	0.8	1.2-5.5
Hypokalemia*^c^*	38.9%	181			
Hypokalemia complications*^d^*	6.7%	31			
Sexual development					
Disordered sexual development	59.5%	278			
Primary amenorrhea	38.3%	178			
Cryptorchidism	15.3%	71			
Atypical genitalia	14.2%	66			
Breast development					
None	64.2%	215			
Incomplete	17.8%	83			
Near complete	3.9%	18			
Complete	0.4%	2			
Breast Tanner stage					
I	48.0%	223			
II	7.3%	34			
III	5.2%	24			
IV	2.6%	12			
V	3.4%	16			
Pubic hair Tanner stage					
I	44.9%	209			
II	8.0%	37			
III	0.9%	4			
IV	1.5%	7			
V	0.4%	2			
Hormone concentrations					
11-deoxycorticosterone (<15 y < 0.9, ≥15 y < 0.3 nmol/L)		80	10.1	14.2	0.0-98.5
Corticosterone (<15 y 0.5-56.8, ≥15 y 1.5-45 nmol/L)		73	593.7	445.6	0.9-1962.9
PRA (<15 y 0.8-3.5, ≥15 y 0.6-4.3 ng/mL/h)		151	0.8	2.0	0.0-18.8
Aldosterone (60-980 pmol/L)		187	611.7	957.9	0.0-8616.0
ACTH (<10 pmol/L)		259	57.6	82.5	0.2-598.8
11-deoxycortisol (0.35-4.56 nmol/L)		38	4.4	6.5	0-29.8
Cortisol (200-650 nmol/L, morning)		301	92.9	113.7	0.0-706.2
17-hydroxyprogesterone (<6 nmol/L)		248	4.8	9.4	0.0-75.0
DHEA-S (nmol/L)*^e^*		186	707.5	3454.1	0.0-37850.0
Androstenedione (nmol/L)*^f^*		148	4.1	45.36	0-429.5
Testosterone (M 8-30, F/PP <2 nmol/L)		215	0.8	3.8	0.0-54.8
Estradiol (M 40-100, F 100-900, PM 70-200 pmol/L)		216	58.6	70.18	0.0-776.5
Progesterone (M < 0.6, F 2-70, PP <1.1 nmol/L)		256	29.9	32.4	0.0-221.2
FSH (M 1-5, F 1-8, PP 0.6-7 mIU/mL)		282	53.5	39.5	0.2-196.8
LH (M 2-10, F 2-15, PM 15-100, PP <11.9 mIU/mL)		282	36.7	42.3	0.0-329.0

Abbreviations: BMI, body mass index; DHEA-S, dehydroepiandrosterone sulphate; F, female; PM, postmenopausal; M, male; PP, prepubertal; PRA, plasma renin activity.

^*a*^Hypertension, systolic blood pressure ≥140 mm Hg.

^*b*^Hypertensive complications include headaches, stroke, and hypertensive retinopathy.

^*c*^Hypokalemia, serum potassium <3.5 μmol/L.

^*d*^Hypokalemic complications include as fainting, muscle weakness, myalgia, muscle spasms, periodic paralysis, and arrhythmias.

^*e*^DHEA-S, M: <15 y 0.15-11, ≥15 y 0.8-20, F: <15 y 0.3-11.7, ≥15 y 1.2-20.7 nmol/L.

^*f*^Androstenedione, M: <15 y 1-3.5, ≥15 y 1.4-5.2, F: <15 y 1.5-6.6, ≥15 y 1-7 nmol/L.

The majority (57.0%, n = 265) of patients were hypertensive, although related complications including headaches, stroke, and hypertensive retinopathy were uncommon (6.7, n = 31). Hypokalemia was reported in 45.4% (n = 211), with 6.7% (n = 31) experiencing symptoms such as fainting, muscle weakness, myalgia, muscle spasms, periodic paralysis, and arrhythmias.

A DSD was present in 59.5% (n = 278) of patients. Common features included cryptorchidism (30.0%, n = 72, of 46,XY patients) and atypical genitalia (14.2%, n = 66). Primary amenorrhea was reported in 38.3% (n = 178) of females.

Hypertension was uncommon in XY males (7.1%, n = 2) but was present in more than half the XX females (57.4%, n = 116) and XY females (63.1%, n = 137) (*P* < .001). Among patients with a potassium assessment, all XY males were normokalemic (5/5) but 65.7% (n = 90) of XY females and 63.2% (n = 84) of XX females (*P* < .05) had hypokalemia.

An absence of pubertal development was reported in 46.2% (n = 215) of the patients, with a further 22.2% (n = 103) having some development, and the remainder being either too young for assessment or puberty was not reported. More than half the XY females (58.0%, n = 123) and XX females (57.5%, n = 88) had delayed puberty, whereas this was reported in fewer (12.5%, n = 1) XY males (*P* < .001; Fig. 3B). Tanner stages are provided in Table 1.

### Hormone Concentrations

Most patients had high serum concentrations of the following hormones: ACTH (88.0%, n = 227), FSH (98.2%, n = 190 of postpubertal patients), and LH (82.5%, n = 175 of postpubertal patients) (Table 1). By comparison, the majority had normal serum concentrations of aldosterone (75.6%, n = 149), 17-hydroxyprogesterone (79.1%, n = 197), and progesterone (91.1%, n = 184 for postpubertal patients). In contrast, a majority of patients had low serum concentrations of cortisol (84.5%, n = 246) and estradiol (85.8%, n = 151 for postpubertal patients). Serum concentrations of testosterone were variable (43.5% normal, 45.7% low or undetectable).

In the majority of patients, serum aldosterone and progesterone levels were withing the normal reference ranges (75.5% and 59.9%, respectively). Most recorded patient serum corticosterone and cortisol values were outside the normal reference ranges, 92.0% high and 84.8% low, respectively. Serum corticosterone to cortisol ratios were elevated in 94.9%.

### Variants

Of the 401 patients with information on genotype, 52.4% (n = 210) had 2 non-null variants (C2), 29.4% (n = 118) had 2 null variants (N2), and 15.7% (n = 73) had 1 null variant and 1 non-null variant (NC). Homozygosity was reported in 65.3% (n = 77) of the N2 group and in 69.7% (n = 145) of those in the C2 category. In 11 cases, only a single variant was identified by contemporary genomic testing.

**Figure 4.**
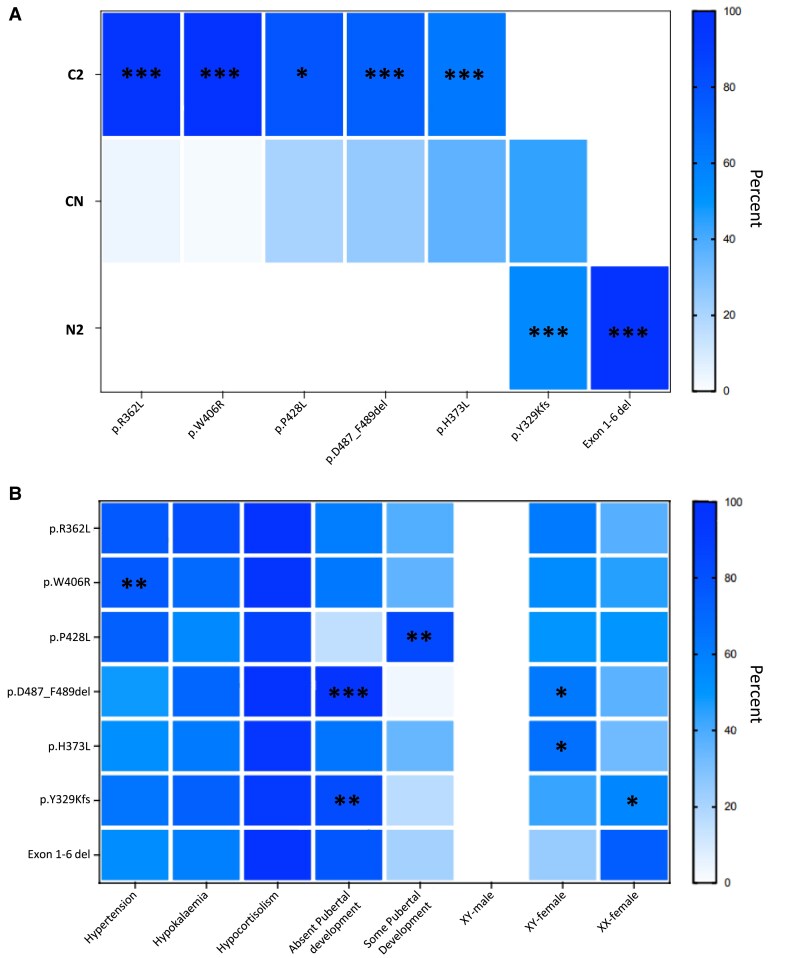
Heatmap representation of genotype-phenotype correlation of XX patients with 17α-hydroxylase/17,20-lyase deficiency. (A) Severity category of prevalent genetic variants. (B) Prevalence of key clinical features in patients with common genetic variants. Significance: *P < .05, **P < .01, ***P < .001. Abbreviations: C, change in sequence variant; N, null variant.

**Figure 5.**
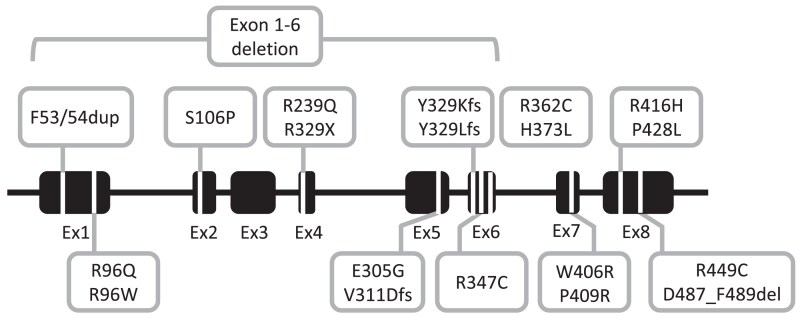
Gene variant diagram for protein level variants reported in 5 or more patients.

The most common variants were p.Y329Kfs (n = 86), p.W406R (n = 39), p.D487_F489del (n = 44), p.H373L (n = 30), p.R362C (n = 21), p.P428L (n = 19), and exon 1-6 deletion (n = 13). The most prevalent non-null variants included p.R362L, p.W406R, p.P428L, p.D487_F489del, and p.H373L (Fig. 4, heatmap A). The most prevalent null variants included p.Y329Kfs and exon 1-6 deletion (heatmap A). Variants reported in 5 or more patients are depicted according to location within the *CYP17A1* gene in Fig. 5.

**Figure 6.**
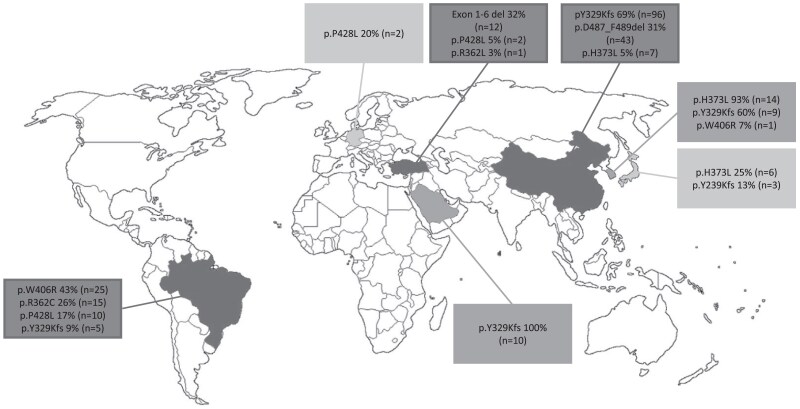
Geographical distribution of most prevalent *CYP17A1* variants by country.

Regional differences in variant distribution included a predominance of p.W406R (42%, n = 27), p.R362L (25%, n = 16), p.P428L (15%, n = 10), and p.Y329Kfs (8%, n = 5) in South/Central America and exon 1-6 deletion (25%, n = 13), p.Y329Kfs (19%, n = 10), and p.P428 (4%, n = 2) in the Middles East. In Asia, the most prevalent variants were p.Y329Kfs (68%, n = 108), p.D487_F489del (28%, n = 44), and p.H373L (17%, n = 27). Country-specific analysis demonstrated the most diverse range of common variants in Brazil, whereas p.Y329Kfs was the only variant among patients from Saudi Arabia (Fig. 6).

### Genotype-phenotype Relationship

The relative presences of hypertension, hypokalemia, hypocortisolemia, and pubertal delay varied between the 3 genotype categories (N2, C2, and NC) (Fig. 3A). Hypertension was more common among patients with at least 1 null variant (60.0% [n = 45] in NC and 67.5% [n = 79] in N2 [*P* < .05]) than in those in the C2 group (50.7% [n = 106]), *P* < .05. Hypokalemia did not differ between the groups, but combined hypertension and hypokalemia was more prevalent in patients in the N2 (65.2%, n = 45) and NC (61.7%, n = 29) groups relative to those in C2 (44.1%, n = 52) (*P* < .01). Absence of pubertal development was identified in 82.9% of N2 patients (n = 68) and in fewer of those in NC (62.3%, n = 33) and C2 (62.9%, n = 88) (*P* < .01).

Hypocortisolemia was common across all genotypes but was more prevalent in patients with at least 1 null variant: 98.6% of N2 (n = 70), 87.5% of NC (n = 49), and 85.7% of C2 (n = 108) (*P* < .05). The ratio of corticosterone to cortisol was higher in patients with no pubertal development (mean ratio, 46.4 [65.5]) compared with patients with some development 11.4 (12.2) (*P* < .01, n = 35). Patients in the NC category had a lower mean ratio (8.1 [6.9]) than those in the N2 (39.6 [36.6]), and C2 (36.8 [64.0]) categories (n = 50, *P* < .01).

The relative presence of clinical features varied according to specific variants (Fig. 4, heatmap B). Some pubertal development was less likely in patients with p.Y329Kfs (17.2%, n = 11; *P* < .01) and p.D487_F489del (3.6%, n = 1; *P* < .001) variants, whereas patients with p.P428L variants were more likely to have undergone some pubertal development (84.6%, n = 11; *P* < .001). Patients with p.W406L variants had a higher prevalence of hypertension (76.9%, n = 30; *P* < .01). There were relatively more XX female patients with p.D487_F489del (62.9%, n = 22; *P* < .05), and p.H373L (66.7%, n = 20; *P* < .05) variant, whereas the p.Y329Kfs group had a higher proportion of XY females (56.5%, n = 48; *P* < .05).

The degree of pubertal development of patients was related to mean diastolic blood pressure, serum potassium, and serum cortisol. Patients with no pubertal development had a higher mean diastolic blood pressure (101.9 [16.8] mm Hg, 96 [17.4] mm Hg respectively; *P* < .05), decreased serum potassium (3.0 [0.7] mmol/L, 3.3 [0.8] mmol/L, respectively; *P* < .01), and decreased serum cortisol (1.6 [0.5] nmol/L, 1.9 [0.5] nmol/L, respectively; *P* < .001) when compared to those with some degree of pubertal development.

## Discussion

This meta-analysis examined the genotypic and phenotypic characteristics of the largest combined dataset of patients with 17-OHD reported in the literature to date. Most (90.8%) patients with this disorder identified as female, although 52.5% had a 46,XY karyotype. Nearly half had 2 non-null variants, whereas one quarter had 2 null variants. Variants ranged from single base substitutions resulting in missense variants (p.R362L, p.W406R, p.P428L, and p.H373L) to multiexon deletions (exon 1-6 del). Most patients had hypocortisolemia, whereas hypertension and DSD were common, and just under half had hypokalemia. Genotype-phenotype analysis demonstrated that patients with 2 null variants had more severe symptoms of 17-OHD. An absence of pubertal development or the combined presence of hypertension and hypokalemia were the clinical parameters that best distinguished severe from milder 17-OHD.

Nearly half (46.2%) the patients of eligible age had no evidence of puberty at presentation, and more than one-third presented for investigation of primary amenorrhoea. Generally, patients with the 46,XX karyotype and 46,XY individuals raised as females presented in later adolescence with pubertal delay. In contrast, 46,XY individuals raised as males typically presented in early childhood for investigation of atypical genitalia and cryptorchidism. Absent pubertal development was associated with other indicators of severe 17-OHD, including elevated blood pressure and marked reductions in serum potassium and cortisol. Conversely, the presence of at least 1 non-null variant was associated with some pubertal development, a lower likelihood of hypocortisolemia and less severe blood pressure elevation. This is consistent with earlier studies and suggests that sexual development is dependent on the least severe allele, whereas the likelihood of hypertension and hypokalemia increase with overall genotype severity (21, 22).

17-OH deficiency, and the associated rise in ACTH because of low cortisol, leads to elevated 11-deoxycorticosterone and corticosterone concentrations. These adrenal biosynthetic intermediates, when in high concentrations, have mineralocorticoid activity, leading to hypertension and hypokalemia, or weak glucocorticoid activity, respectively (23). In this study, hypocortisolemia was found in the majority (85%) of patients but adrenal crises were rare, seen in only 2 patient histories. This is likely because of the glucocorticoid effect of corticosterone (23). Episodes of severe adrenal insufficiency, however, may be more likely after treatment of affected individuals because this reduces ACTH and corticosterone concentrations, increasing the severity of endogenous glucocorticoid deficiency. Although there was no correlation between 11-deoxycorticosterone and hypokalemia in this study, the presence of more severe (null) *CYP17A1* variants was associated with hypertension and hypokalemia, likely because of more impaired 17-OHase activity.

The geographic distribution of reported cases of 17-OHD differed to that anticipated by factors such as population size and health care accessibility, with China, Brazil, and the Middle East being the predominant origins, possibly resulting from founder effects or consanguinity. Geographically recurring variants indicating a founder effect have been reported previously in the Frisian (p.P480H) and Chinese (p.Y329 K, p.R362C, and p.P328L) populations (10). The present analysis supports the presence of a founder effect for p.Y329Kfs and p.D487_F489del variants in China, which is consistent with previous evidence (24). The exon 1-6 variant, identified as a founder variant in eastern Turkey, was found almost entirely (92%) in homozygotes in this study, supporting consanguinity as a causal factor (25, 26). By comparison, variants p.W406R, p.R362C, and p.428L were more prevalent among Brazilian patients, reflecting the higher prevalence of 17-OHD in South America, particularly Brazil (8, 10, 27, 28).

17-OHD typically leads to low levels of cortisol and sex steroids, although progesterone may be elevated in some patients. Elevated progesterone combined with low cortisol was proposed as a simple and reliable criterion for early workup of possible cases of 17-OHD based on an analysis of 150 46,XY patients (10). Although the present study corroborated the correlation between variant severity and cortisol level, this did not apply to levels of progesterone, 11-deoxycortisol, or corticosterone (10). Possible reasons for this discrepancy include limitation of the initial sample to 46,XY individuals and incomplete data in case reports, as detailed hormonal assays were rarely available (8.2% had 11-deoxycortisol and 10.7% had a corticosterone level assessed) for analysis in the present study.

Only 15% to 20% of patients with 17-OHD were found to manifest simultaneously the classical triad of hypertension, hypokalemia, and DSD (27). As a consequence, it is not surprising that misdiagnosis and delayed diagnoses are common. Earlier diagnosis is more frequent in 46,XY individuals because of undervirilization of the external genitalia and consequent presentation with atypical or visually female genitalia and cryptorchidism (29). In comparison, 46,XX patients have no apparent genital anomalies at birth, and are typically diagnosed later than 46,XY individuals, regardless of variant severity. Primary amenorrhea affected 58.3% of the 46,XX patients aged 15 years or more, demonstrating the importance of considering 17-OHD as a differential diagnosis for patients presenting with primary amenorrhea and hypertension.

This study reports a comprehensive examination of the largest cohort of patients with 17-OHD to date. It was, however, limited by missing data and variability in hormone assays, reporting units, and age at clinical and biochemical evaluations. Although care was taken to include individual patients only once, duplications are possible. Some patients could not be included in the analysis because raw data were not available from some large studies (12). Other constraints included inherent limitations in genotyping, such as changes to sequencing methodologies, genome builds, and reporting styles over time. Unavailability of raw sequencing data prevented full variant labelling and classification according to current guidelines. Homozygosity was assumed for some cases because of reporting of a single variant only, but these may have had an identifiable heterozygous variant or a second variant that could not be detected by the genetic testing methodologies available at that time. This limitation may have led to misclassification of the genotype category in a small number of patients. In addition, compound heterozygosity was assumed when 2 variants were detected, but whether these variants were truly *in trans* (resulting in biallelic inactivation/impaired function) was generally not evident. It is also possible that some missense variants (classified as non-null variants) completely abolish enzymatic function and would be better considered equivalent to a null variant. In addition, the C group category of cases in this analysis may have been enriched with missense variants that have a null effect, for example the p.H373L and p.W406R variants that have been shown to have no enzymatic function, but according to the classification were included in the “C” category (30). Identification of partial deficiency requires an enzymatic defect of sufficient severity and, therefore, patients with enzymatic function >25% might remain unidentified. This may have affected adversely determination of the complete spectrum of the 17-OHD genotype phenotype relationship in this analysis (30).

In conclusion, 17-OHD is a frequently underdiagnosed disorder because of its relative rarity and variable clinical presentation. Patients typically present as females with primary amenorrhea and hypertension or as males with atypical genitalia or cryptorchidism. Because of the absence of overt genital abnormalities, females are typically diagnosed later than males. Genotype-phenotype correlations are evident, with null variants being more commonly associated with absent pubertal development, hypertension, combined hypertension and hypokalemia, and hypocortisolism than non-null variants. Ongoing research using the latest sequencing technologies and reporting systems as well as parental testing or long-read sequencing for variant segregation and enzymatic studies (particularly of missense variants) will further clarify the relationship between *CYP17A1* variants and phenotypes.

## Data Availability

Deidentified patient data will be made readily available after publication upon request to the corresponding author for research purposes to qualified individuals within the scientific community.

## Abbreviations

17-OH: 17α-hydroxylase/17,20-lyase
17-OHD: 17α-hydroxylase/17,20-lyase deficiency
C2: 2 non-null variants
DSD: disordered sexual development
N2: 2 null variants
NC: 1 null variant and 1 non-null variant
